# Differential Effects of Cognitive vs. Motor Dual-Task Training in Stroke Rehabilitation: A Precision-Focused Meta-Analysis

**DOI:** 10.3390/geriatrics11030065

**Published:** 2026-05-31

**Authors:** Hui Gao, Man Lang, Mustapha Mangdow, Wen Liu

**Affiliations:** 1 Department of Physical Therapy, Rehabilitation Science, and Athletic Training, School of Health Professions, University of Kansas Medical Center, Kansas City, KS 66160, USA; hgao3@kumc.edu (H.G.); mmangdow@kumc.edu (M.M.); 2 Shanghai YangZhi Rehabilitation Hospital (Shanghai Sunshine Rehabilitation Center), School of Medicine, Tongji University, Shanghai 201619, China; langmanzaszaszsu@163.com

**Keywords:** dual-task training, stroke, precision medicine, geriatric neurology

## Abstract

Objectives: Stroke predominantly affects older adults and is accompanied by age-related declines in balance and mobility. Given the inter-individual variability in post-stroke functional capacity, identifying the most effective dual-task training modalities for specific patient profiles is essential for precision-based stroke rehabilitation. This systematic review and meta-analysis aimed to investigate the differential effectiveness of motor dual-task training (MDT) and cognitive dual-task training (CDT) on gait performance, balance control, and motor function while exploring other moderating factors. Methods: The study followed the PRISMA guidelines. A comprehensive search of six English databases (Web of Science, PubMed, Medline, Embase, Cochrane Library, CINAHL) and one Chinese database (CNKI) was conducted from November 2023 to June 2025. Randomized controlled trials involving adult stroke survivors were included. Outcomes included gait, balance, and lower-extremity motor function. Random-effects meta-analysis, subgroup analyses, and meta-regression were performed to evaluate modality-specific effects and moderating factors. Results: Twenty-one RCTs (*n* = 786) were included. Dual-task training demonstrated moderate improvements in both temporal (SMD = 0.50, *p* = 0.03) and spatial (SMD = 0.5, *p* = 0.04) gait performance and balance control (SMD = 0.71, *p* = 0.02), but not motor function. MDT demonstrated superior effects on gait speed and stride length (SMD = 1.15, *p* = 0.01; SMD = 0.89, *p* < 0.01), whereas CDT showed greater benefits for balance (SMD = 0.59, *p* < 0.01). Greater balance improvements were observed in individuals at high fall risk, and subacute patients showed enhanced responsiveness. Conclusions: These findings provide guidance for tailoring dual-task modality and timing to individual patient profiles, though high heterogeneity and the lack of direct comparisons between MDT and CDT limit definitive conclusions.

## 1. Introduction

Stroke is one of the leading causes of disability [1] and disproportionately affects older adults, who often present with complex clinical profiles including frailty, cognitive decline, and multimorbidity. In geriatric populations, stroke-related impairments in gait pattern, trunk posture, and balance control, as well as cognitive and perceptual function, are particularly pronounced [2]. Balance control is the ability to maintain body movement within the base of support without falling, which can be influenced by cognitive factors, such as attention, motivation, and intent [3]. Gait impairment consists of slow walking speed and short duration, and affects activities of daily living (ADLs) in stroke survivors [4]. Most ADLs require the ability to perform two or more tasks simultaneously [5]. Therefore, dual-task training involving performing two or more tasks at the same time has been widely applied and evaluated in stroke rehabilitation [6].

However, the evidence supporting the benefits of dual-task training in improving overall physical function after stroke remains mixed. This inconsistency may be due to the way that dual-task training is often treated as a single, uniform intervention, despite wide variation in both patient characteristics and training methods. A meta-analysis [7] conducted in 2021 indicated significant benefits of dual-task training on balance, gait, and upper limb function compared with conventional therapy, whereas a 2022 meta-analysis [6] reported no significant difference on balance outcome between the comparative groups. Dual-task studies are often classified into two different types: cognitive dual tasks (CDTs) or motor dual tasks (MDTs), which respectively refer to the simultaneous performance of one cognitive task and one motor task [8] or the simultaneous performance of two different motor tasks [9]. Only two past studies directly compared two different types of dual-task training. Inconsistent findings were reported of either better outcomes after the CDT program than the MDT program [10] or the opposite [11]. CDTs and MDTs differ in their underlying mechanisms and clinical application targets. CDTs are thought to target attentional resource allocation and cognitive-motor integration, which challenges the prefrontal cortex-mediated executive function and attention systems [12]. In contrast, MDTs place greater demands on sensorimotor coordination, neuromuscular control, and the integration of multiple motor programs [13]. Therefore, analyzing their effects separately are warranted to inform more targeted, evidence-based rehabilitation prescriptions. Furthermore, patient characteristics such as stroke chronicity and baseline function, both of which are known to influence treatment response, were not accounted for in prior meta-analyses.

Aligned with the NIH milestones for advancing precision medicine in rehabilitation [14], this systematic review and meta-analysis is structured around three objectives. The primary objective is to examine the differential effectiveness of two dual-task training modalities, CDT and MDT, on gait, balance control, and motor function in stroke survivors. Given the heterogeneity of post-stroke functional impairments in the aging population, clarifying these differential effects may facilitate personalized prescription of dual-task training tailored to specific functional deficits, thereby moving beyond a “one-size-fits-all” rehabilitation approach. The second objective is to explore potential moderators of training effects, including participant characteristics and types of control interventions. Stroke survivors with different levels of chronicity or baseline functional impairment severity in walking or balance control may respond differently to dual-task training. Investigating these moderating factors can further support the implementation of dual-task interventions within a precision medicine framework. Finally, by including several newly published clinical trial reports [15,16,17,18,19] of dual-task training, this review and meta-analysis study provides an updated quantitative synthesis of the overall effects of dual-task training in stroke rehabilitation.

## 2. Materials and Methods

This study was performed and reported according to the Preferred Reporting Items for Systematic Reviews and Meta-Analysis (PRISMA) guidelines [20]. The study protocol was registered in the International Prospective Register of Systematic Reviews (PROSPERO) with registration number CRD42023477417. https://www.crd.york.ac.uk/PROSPERO/view/CRD42023477417 (accessed on 1 April 2026).

### 2.1. Search Strategy

A comprehensive and systematic literature search was conducted independently by two authors (HG and ML) across six English databases (Web of Science, PubMed, Medline, Embase, Cochrane Library, CINAHL) and one Chinese database (Chinese National Knowledge Infrastructure). The search strategy was developed using a combination of MeSH terms and free-text keywords related to stroke (e.g., “cerebrovascular accident”, “cerebral stroke”, “brain vascular accident”, “CVA”, “cerebrovascular apoplexy”) and dual-task (e.g., “dual-task exercise”, “dual-task interference”, “dual-task performance”, dual-task training”, “dual-task practice”). Boolean operators (AND/OR) were used to structure search strings, with database-specific syntax adjustments. Filters were applied to restrict results to human studies involving participants aged ≥50 years. The complete electronic search strategy for all databases is available in Supplementary Table S1. The literature search commenced in November 2023 and concluded in June 2025.

### 2.2. Inclusion and Exclusion Criteria

The inclusion and exclusion criteria for this study were defined using the PICOS (Participant, Intervention, Comparisons, Outcomes, and Study Design) framework to ensure methodological rigor and relevance to the research objectives. The inclusion criteria for study selection were (1) participants who were stroke survivors and aged above 50 years old to ensure an aging population; (2) cognitive dual-task training or motor dual-task training; (3) control group received standard treatment or no treatment; (4) outcomes included one or more of the following: temporal gait parameter (gait speed), spatial gait parameter (stride length), balance (Berg Balance Scale (BBS)), and motor function (Fugl–Meyer lower extremity assessment score (FMA_LE)); (5) randomized controlled trials that used dual-task training compared with conventional therapy or sham control; (6) full-text studies published in English, or Chinese articles with an English abstract. Exclusion criteria were (1) healthy population or non-stroke patients as comparative participants; (2) dual-task training combined with other confounding training such as aquatic exercise; (3) protocols, reviews, letters, academic theses, and congress abstracts; (4) studies where the outcome data (e.g., mean ± SD) could not be extracted from the text, tables, or figures, and author contact was unsuccessful.

### 2.3. Data Collection and Quality Evaluation

Full-text articles meeting the initial screening criteria were independently reviewed by two investigators against the predefined inclusion and exclusion criteria, with disagreements resolved by consensus, and when necessary, adjudication by a blinded third reviewer. After two researchers screened the literature and identified a final list of qualified studies, data extraction was performed according to the Cochrane Handbook for Systematic Reviews of Interventions [21]. The following data were extracted: sample size, patients’ characteristics, intervention characteristics, and outcomes. To facilitate the subgroup analyses, patient characteristics such as fall risk and walking ability were defined using baseline scores from the BBS and gait speed, respectively. Fall risk was categorized based on BBS scores as follows: low risk (41–56), moderate risk (21–40), and high risk (0–20) [22]. Walking ability was assessed using comfortable gait speed, with 0.49 m/s serving as the threshold to distinguish between home ambulators and community ambulators [23]. In terms of the intervention characteristics, dual-task training was categorized into CDTs and MDTs. The type of basic motor training was classified as gait training, balance training, or mixed training. For instance, if the basic motor component of dual-task training involved walking, the intervention was categorized as dual-task gait training. The types of control were classified as either active or passive. Active control means comparable standard treatment, and passive control refers to no additional intervention other than usual care. Methodological rigor and potential biases were also documented to ensure systematic data collection and analysis.

The risk of bias of the studies was appraised using the Physiotherapy Evidence Database (PEDro) scale. Studies with a score of six or above were classified as low risk, whereas scores of four and five were classified as fair risk, and scores below three were considered high risk [24]. Discrepancies that could not be resolved through discussion were evaluated by a third expert. Additionally, the overall certainty of the evidence for each outcome was graded using the GRADE [25] (Grading of Recommendations, Assessment, Development, and Evaluations) framework. This tool assesses the study limitations, inconsistency, indirectness, imprecision, and publication bias. This comprehensive evaluation ensured the reliability and interpretability of the synthesized evidence.

### 2.4. Statistical Analysis

StataSE (15.1) was used to conduct meta- and subgroup analysis on three outcomes: gait performance (gait speed and stride length), balance (BBS), and motor function (FMA_LE). After completing the analysis, relevant values for each parameter, such as *p*-values, standardized mean differences (SMD), etc., were entered into Microsoft Excel to create a visually appealing forest plot that summarizes the descriptive data [26]. Heterogeneity was assessed using the I^2^ statistic, with the following interpretation: 0–40% low heterogeneity, 30–60% moderate heterogeneity, 50–90% substantial heterogeneity, and 75–100% considerable heterogeneity [21]. When an I^2^ value was greater than 50%, a random-effects model was applied; otherwise, a fixed-effects model was used. Pooled effect sizes were calculated with z tests, where a *p*-value < 0.05 demonstrated statistical significance. We used Hedges’ g with 95% confidence interval (CI) to calculate effect sizes, as it corrects for small sample sizes, which were categorized as follows: small (≥0.2), medium (≥0.5), and large (≥0.8) [27].

Comprehensive subgroup analyses were conducted to examine the differential effectiveness of the dual-task training. Key comparisons included the training modalities (CDT vs. MDT), stroke chronicity (<6 months vs. ≥6 months), fall risk levels (low vs. moderate vs. high), walking ability (home ambulator vs. community ambulator), type of basic motor training (gait training vs. balance training vs. mixed training), and type of control method (active vs. passive control). To ensure statistical robustness, any subgroup containing only a single comparison or insufficient data to calculate heterogeneity or *p*-values was excluded. Furthermore, we conducted meta-regression on the dual-task modalities (CDT vs. MDT) to statistically justify the differential findings observed in the subgroup analysis (results are presented in Appendix A). Sensitivity analyses were performed to assess the stability of the primary findings and identify studies disproportionately contributing to heterogeneity. To assess potential publication bias, Egger’s regression test was conducted. This is a quantitative test with *p* < 0.05 indicating significant publication bias.

## 3. Results

### 3.1. Study Selection

**Figure 1:**
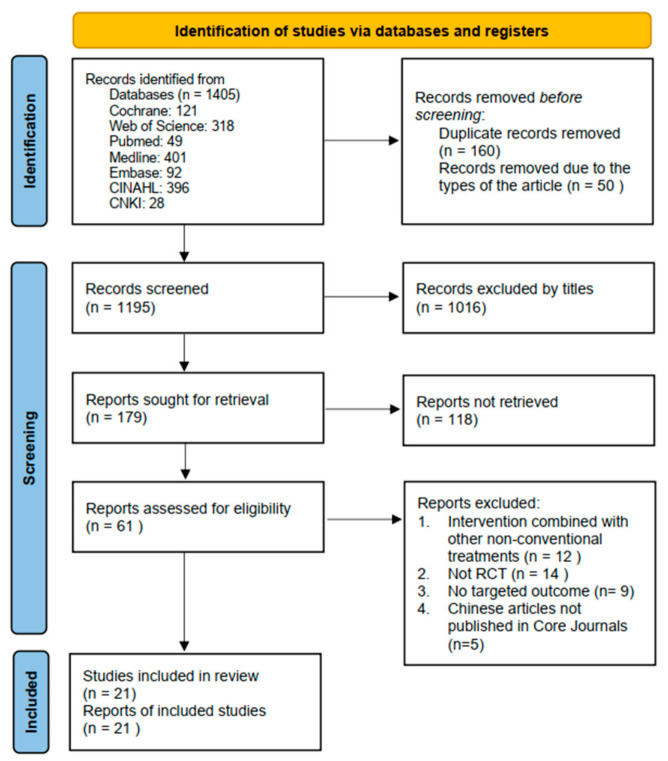
PRISMA flow diagram.

Figure 1 illustrates the literature search process. An initial search identified 1405 articles. After removing duplicates, systematic reviews, and meta-analyses, 1195 articles remained and were screened for eligibility by title. Of these, 118 were excluded based on abstract review. Finally, 21 studies were included after full-text review. Of those studies, three [28,29,30] were in Chinese and 18 [9,10,13,15,16,17,18,19,31,32,33,34,35,36,37,38,39] were in English.

### 3.2. Study Characteristics

These studies were published between 2007 and 2025, and the study locations included the USA, Pakistan, Turkey, South Korea, Taiwan, and China. A total of 786 participants were included in this review, with 396 participants allocated to the dual-task group and 390 to the control group. None of the included studies reported significant baseline differences between comparative groups in terms of gender distribution, age, or disease duration. In most studies, there were no significant baseline differences in outcome measures between the control and experimental groups. In only one study [33], the baseline stride length of the control group was almost twice that of the experimental group. The detailed characteristics of the participants and intervention of the included studies are summarized in Table 1 and Table 2, respectively.

### 3.3. Risk of Bias and GRADE Assessment

The final PEDro score ranged from five to eight points, with 80.96% of the included studies [9,10,13,17,18,19,28,30,31,32,33,34,35,36,37,38,39] being low risk, scoring ≥ 6/10, and 19.04% of the included studies [15,16,29,39] being fair risk (Table 3).

The GRADE assessment of the included studies predominantly revealed a moderate to high certainty of evidence across all four outcomes. Gait speed and stride length often achieved high certainty in trials utilizing objective measurement tools like instrumented walkways and prospective registration, though some studies were downgraded due to small sample sizes [10,15]. FMA_LE and BBS typically ranged from moderate to high certainty, with common downgrades stemming from a serious risk of detection bias in studies where outcome assessors were not blinded to the treatment groups [29,38]. A detailed summary of the GRADE ratings for each outcome is presented in Supplementary Table S2.

### 3.4. Meta-Analysis and Meta-Regression

#### 3.4.1. Gait Performance

**Figure 2:**
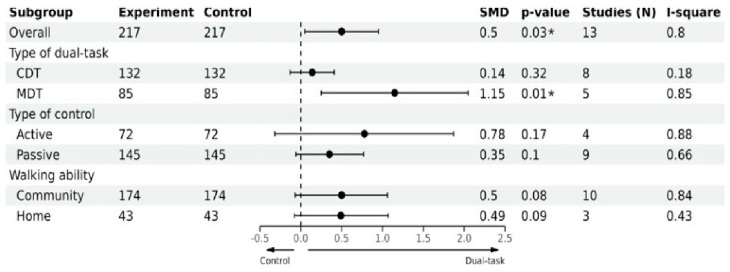
Gait speed. SMD: Standardized mean difference; *: *p*-value < 0.05.

**Figure A1:**
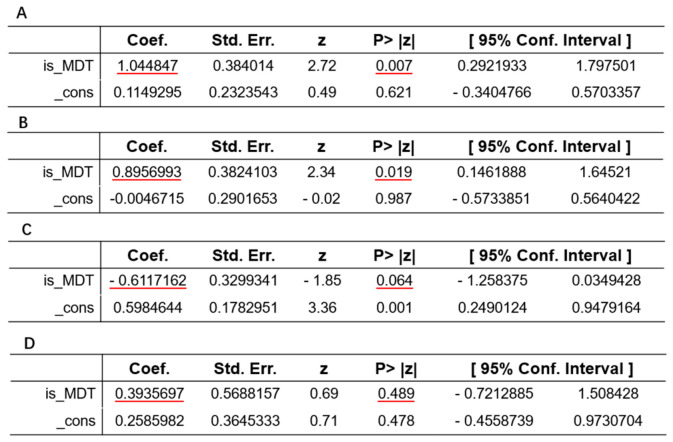
Random-effects meta-regression analysis comparing the moderation effects of MDT versus CDT across clinical outcomes. (**A**) Gait speed and (**B**) stride length, showing significant differential effects favoring MDT over CDT (*p* = 0.007 and *p* = 0.019; Coef. = 1.04 and Coef. = 0.90). (**C**) Berg Balance Scale (BBS), demonstrating a non-significant trend favoring CDT over MDT (*p* = 0.64; Coef. = −0.61). (**D**) Fugl–Meyer Assessment for Lower Extremity (FMA_LE), indicating that intervention modality was not a significant moderator of motor recovery. The red lines were included for illustration purposes.

Twelve studies [9,10,13,15,16,17,18,19,33,34,35,37] reported gait speed as the temporal gait parameter. One [10] study included both MDT and CDT as intervention groups, allowing for two separate comparisons within the same study. Therefore, a total of 13 comparisons were made between the experimental group and control group with considerable heterogeneity (I^2^ = 80%). Figure 2 shows a medium overall beneficial effect of dual-task training on gait speed compared to the control group (SMD = 0.5, 95% CI = 0.05–0.95, *p* = 0.03). Due to limited comparisons or the presence of only a single category in some subgroups, subgroup analyses were not conducted for chronicity, basic motor training, or fall risk. Specifically, nearly all included studies recruited chronic patients; only two [16,17] out of twelve studies did not involve gait training, and only five out of the twelve studies reported measures of the BBS. In the subgroup analysis of walking ability, no significant differences were observed between the experimental and control groups for either home ambulators or community ambulators. Similarly, no significant subgroup differences were found based on the type of control. CDT did not demonstrate a significant advantage over conventional training in gait speed (SMD = 0.14, *p* = 0.32). In contrast, MDT showed a significant improvement in gait speed compared to the control groups, with a large effect size (SMD = 1.15) and a *p*-value of 0.01. The mean difference between the experimental and control groups in the MDT subgroup was 0.18 m/s, exceeding the minimal clinically important difference (MCID) for comfortable gait speed (0.16 m/s) [40]. This differential effect between MDT and CDT was also statistically supported by the meta-regression (*p* = 0.007), with a positive coefficient of 1.04 confirming the superior efficacy of MDT (see Figure A1 in Appendix A).

**Figure 3:**
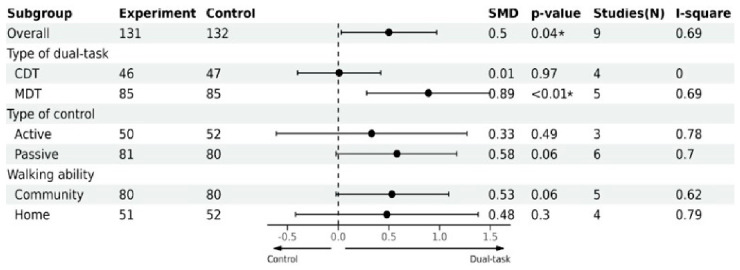
Stride length. SMD: Standardized mean difference; *: *p*-value < 0.05.

As shown in Figure 3, eight studies [9,10,13,19,31,33,34,35,37] were included, and nine comparisons were made to assess the effect of dual-task training on stride length. One study [33] was excluded from the analysis due to substantial baseline discrepancies. The overall impact of dual-task training on stride length demonstrated a moderate effect with substantial heterogeneity (SMD = 0.5, 95% CI = 0.03–0.97, *p* = 0.04, I^2^ = 69%). In the subgroup analysis, MDT demonstrated a large effect size (SMD = 0.89, *p* < 0.01), whereas CDT showed no significant difference compared to the control group (SMD = 0.01, *p* = 0.97). Meta-regression analysis provided statistical support for dual-task type significantly moderating the treatment effect (*p* = 0.019), confirming that MDT demonstrated a statistically superior benefit, associated with a 0.90 larger effect size than CDT (see Figure A1 in Appendix A). In the subgroup analysis by control type, neither the active control group (*p* = 0.49) nor the passive control group (*p* = 0.06) showed statistically significant differences between the experimental and control conditions. Likewise, in the walking ability subgroup analysis, no significant effects were observed for community ambulators (*p* = 0.06) or home ambulators (*p* = 0.30).

#### 3.4.2. Balance Control

**Figure 4:**
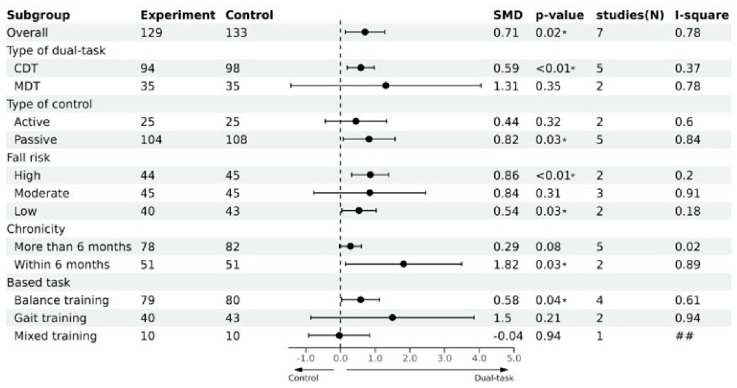
Berg Balance Scale. SMD: Standardized mean difference; *: *p*-value < 0.05; ##: unable to be calculated due to only one comparison in this subgroup.

Seven studies [29,30,31,32,36,38,39] with significant heterogeneity (I^2^ = 78%) were included and compared the effect of dual-task training on balance control (Figure 4). The combined results of the random-effect model were as follows: SMD = 0.71, 95% CI = 0.14–1.28, *p* = 0.02, indicating a medium beneficial effect of dual-task training. The mean difference between the experimental group and the control group was 4.53, exceeding the MCID of Berg Balance Scale [41]. In the subgroup analysis, patients with high fall risk showed greater improvement following dual-task training (SMD = 0.86, *p* < 0.01) compared to those with low fall risk (SMD = 0.54, *p* = 0.034), while no statistically significant effect was observed in patients with moderate fall risk (SMD = 0.84, *p* = 0.31). A significant training effect on BBS was observed in patients within 6 months post-stroke (SMD = 1.82, *p* = 0.034). This effect was not statistically significant in patients more than 6 months post-stroke (SMD = 0.29, *p* = 0.08). Studies using CDT demonstrated a statistically significant improvement in balance control with a medium effect size (SMD = 0.59, *p* < 0.01). This training effect was not found in studies using MDT (SMD = 1.31, *p* = 0.35). Although the difference in effectiveness between CDT and MDT did not reach statistical significance in the meta-regression analysis (*p* = 0.064), the magnitude and direction of the effect sizes still suggest a preferential trend favoring the use of CDT for enhancing balance control (see Figure A1 in Appendix A). Compared to gait training and mixed training, balance training (SMD = 0.579, *p* = 0.038) was the only one showing a significant difference between the experimental group and control group.

#### 3.4.3. Motor Function

**Figure 5:**
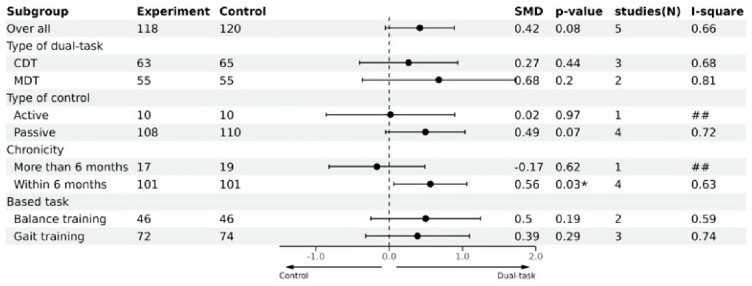
FMA_LE. SMD: Standardized mean difference; *: *p*-value < 0.05; ##: unable to be calculated due to only one comparison in this subgroup.

As shown in Figure 5, five studies [3,18,28,29,30] included FMA_LE as an outcome. No significant beneficial effect of dual-task training on motor function was found (SMD = 0.42, 95% CI = −0.05–0.89, *p* = 0.08, I^2^ = 66%). Meta-regression analysis also did not find a statistically significant difference between CDT and MDT (*p* = 0.49), with a coefficient of 0.50 (see Figure A1 in Appendix A). However, the subgroup analysis based on stroke chronicity again yielded a crucial finding for intervention timing: only patients in the subacute phase demonstrated a significant training effect (SMD = 0.56, *p* = 0.03). No significant difference between the experimental group and control group was found in other subgroup analyses.

### 3.5. Publication Bias

Egger’s regression test indicated no significant evidence of publication bias across any of the main functional outcomes. The *p*-values for gait speed, stride length, BBS, and FMA_LE were 0.97, 0.39, 0.61, and 0.97, respectively, all well above the significance threshold of *p* < 0.05 (see Table A1 in Appendix A).

### 3.6. Sensitivity Analysis

**Figure A2:**
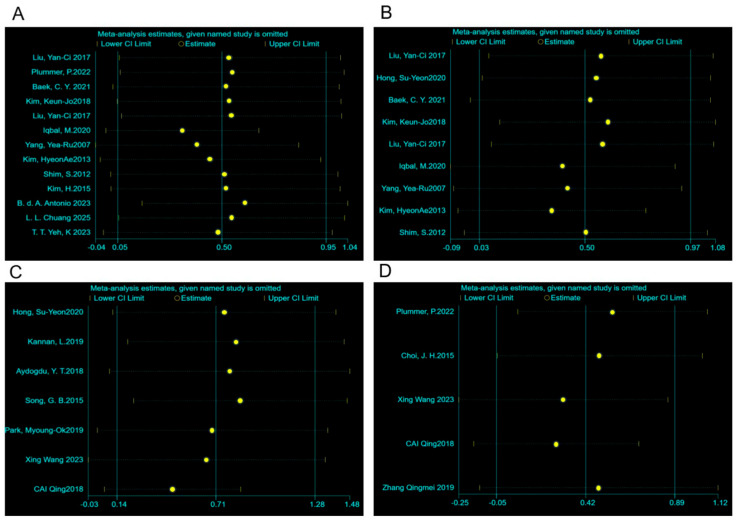
Sensitivity analysis: (**A**) Gait speed; (**B**) stride length; (**C**) Berg Balance Scale; (**D**) FMA_LE. All individual study estimates remained within the 95% confidence intervals of the pooled effect size, indicating that the results were robust and not significantly influenced by any single study. Studies included in (**A**): refs. [9,10,13,15,16,17,18,19,33,34,35,37]; (**B**): refs. [9,10,13,19,31,34,35,37]; (**C**): refs.[29,30,31,32,36,38,39]; (**D**): refs.[3,18,28,29,30].

The stability of the overall findings was confirmed through the sensitivity analysis using the leave-one-out method. Across all outcomes, none of the calculated pooled effect sizes fell outside the 95% confidence intervals, indicating that the overall findings of this meta-analysis are relatively stable and not influenced by any single study (see Figure A2 in Appendix A).

## 4. Discussion

The current meta-analysis systematically investigated the effects of dual-task training on physical recovery among aging stroke survivors at gait performance, balance control, and motor function, focusing on various factors that may influence the effectiveness of dual-task training including the type of dual-task intervention, patient characteristics, and the underlying motor task. The primary finding confirmed that dual-task intervention offers a median effect over conventional treatment in improving both gait performance and balance control, though a consistent benefit was not observed for motor function. More importantly, the results provide clear guidance for a targeted prescription based on patients’ functional goals. MDT was found to be more effective than CDT for enhancing gait performance, whereas CDT seemed to be better than MDT for improving balance control. Furthermore, the patients’ characteristics may also contribute to the effectiveness of dual-task training. Patients within 6 months after stroke were more likely to gain improvement in balance control and physical function. Additionally, dual-task training had a greater effect on balance control in patients with a high risk of falls.

Three previous meta-analysis studies [6,7,42] reported similar improvements on temporal gait parameters such as gait speed and cadence, which were consistent with our findings. There was mixed evidence in terms of spatial gait parameters such as stride length. Two of those studies [6,42] found a beneficial effect of dual-task training on spatial gait performance, which was also supported by our findings. The other one [7] reported no significant difference in stride length. The discrepancy may be attributed to the difference in inclusion criteria of the third study, as it included only studies employing CDT. However, none of those previous meta-analyses specifically examined the differences in effectiveness between CDT and MDT. Only a limited number of studies have explored this topic, but their findings have been inconsistent [10,11,43]. It has been speculated that different types of dual-task training might all positively impact walking performance, but differently influence the walking characteristics [10]. However, in the current study, we found significant effects only by MDT on both spatial and temporal gait parameters with a large effect size, whereas CDT did not demonstrate a significant influence on either parameter. Moreover, MDT was the only subgroup in the meta-analysis of gait performance to show a statistically significant difference among all subgroup analyses. These findings suggest that MDT may present significant training-specific effects, especially since the gait outcomes were single-task measures that did not involve simultaneous cognitive demands. One thing worth mentioning is that in the meta-analysis of gait performance, participants of nearly all included studies were chronic stroke survivors. Although no effect of CDT on gait performance was observed, it remains unclear whether CDT might improve gait performance in subacute stroke survivors. The superior effect of MDT on gait performance may be explained by the principle of task-specificity and the neuromuscular demands inherent to motor–motor dual-task paradigms. When patients simultaneously perform two motor tasks, such as walking while carrying an object or coordinating upper- and lower-limb movements, the neuromuscular system is challenged to execute and integrate multiple motor programs in parallel. This places direct demands on sensorimotor coordination, inter-limb motor planning, and the regulation of muscle synergies, all of which are critical determinants of gait speed and stride length [19].

There have been reports of mixed results of dual-task training on balance control in stroke survivors. One study did not observe the effect of dual-task training on balance control [3], while other studies [31,38,44] reported the superiority of dual-task training over conventional therapy. However, only one of the past three meta-analyses [7] suggested the beneficial effect of dual-task training on balance control, and not in the other two meta-analysis studies [6,42]. Our findings support the overall beneficial effect of dual-task training on balance control in stroke survivors. Furthermore, we found that CDT provided greater improvements for balance control than MDT. The preferential effect of CDT on balance control may be explained by the cognitive-motor interdependence underlying postural stability in stroke survivors. Maintaining balance is not a purely motor task; it requires continuous attentional monitoring, sensory reweighting, and executive control to make rapid postural adjustments in response to perturbations. CDT, which pairs balance or standing tasks with concurrent cognitive demands such as counting or verbal fluency, directly trains the ability to allocate attentional resources to postural control under conditions of divided attention. Moreover, this lack of observed effect of MDT on balance control may also be attributed to the limitations of the BBS assessment, which does not adequately assess dynamic balance control as opposed to the Dynamic Gait Index (DGI) [45]. This is particularly relevant given that MDT is a more dynamic form of training. In subgroup analyses, we found that only balance training combined with a secondary task had a significant effect on balance control, whereas gait training and mixed training did not. This finding is reasonable, as it reflects the principle of task-specificity in training. The characteristics of patients could also influence the effectiveness of dual-task training, since stroke survivors with high fall risk showed the highest level of improvement in balance control, but participants with moderate fall risks did not show significant improvement. This result might be explained by the high heterogeneity of this subgroup (I^2^ = 91%) compared to high fall risk (I^2^ = 20%) and low fall risk (I^2^ = 18%) subgroups. These findings collectively emphasize that for optimizing balance recovery, both the training modality (CDT) and the patient’s baseline fall risk profile must be carefully considered. With more studies included in this meta-analysis, our results confirmed the finding of a previous meta-analysis [6] that no superiority of dual-task training over conventional intervention on lower-extremity motor function. However, subgroup analysis provided a crucial prescriptive insight regarding the optimal timing of intervention. A significant benefit was only found in patients within 6 months post-stroke. This suggests that individuals in the subacute stage of recovery possess a higher level of neuroplasticity or residual function that makes them more responsive to the demands of dual-task interventions for improving lower-extremity motor function.

To the best of our knowledge, the current study is the first meta-analysis to perform subgroup analysis on the differential effects of CDT and MDT as well as other factors that may influence the effectiveness of dual-task training. Such information can contribute to the goal of establishing targeted training protocols for stroke survivors, enabling clinicians to match the intervention modality to specific functional goals [10]. In addition, we included studies published in both English and Chinese. Only Chinese studies published in core journals were included to ensure the quality of the reviewed articles. Furthermore, the methodological integrity of our results was affirmed through sensitivity analysis. Despite moderate to substantial heterogeneities, the overall and subgroup findings for each outcome remained relatively stable when using the leave-one-out method, ensuring confidence in our interpretations.

This study also has several limitations. First, the beneficial effect of dual-task training should be interpreted with caution due to the heterogeneity of the reviewed trial studies. Second, the strength of evidence from subgroup analysis was limited by the small number of included studies. Additionally, the methodology of subgroup analysis did not allow for direct comparisons between categories. Therefore, the observed advantages of MDT over CDT in improving gait performance, and of CDT over MDT in enhancing balance, cannot be conclusively confirmed. More studies directly comparing these two types of dual-task training are needed to strengthen the evidence.

## 5. Conclusions

The effectiveness of dual-task training in stroke rehabilitation is not uniform, particularly given the heterogeneity of functional profiles across the post-stroke population. In stroke survivors aged 50 years and older, where impairments in mobility, balance, and cognition frequently coexist, a precision-based approach to rehabilitation is essential. Our review and meta-analysis investigated the effects of different types of dual-task training, along with other contributing factors such as patient characteristics. MDT appeared to produce greater benefits for gait performance, which was not observed with CDT. Conversely, CDT seemed to be more effective than MDT in enhancing balance function. The effectiveness of dual-task training also varied based on patient characteristics; subacute stroke survivors were more likely to experience improvements in balance control and motor function compared to those at the chronic stage, and stroke survivors with a high risk of falls showed greater gains in balance control. These findings offer prescriptive insights that may guide clinicians in selecting the appropriate dual-task modality and intervention timing based on individual functional profiles. However, the high heterogeneity among studies and the lack of direct comparisons between CDT and MDT limit the strength of these conclusions. Future research should aim to further clarify the specific benefits of different types of dual-task training and identify the most suitable populations to maximize therapeutic outcomes.

## Figures and Tables

**Table 1:** Participant characteristics of the included studies.

	No. of Participants		Age (y), Mean ± SD		Gender (Male/ Female)		Time Since Stroke (m), Mean		Fall Risks	Walking Ability
Study	EG	CG	EG	CG	EG	CG	EG	CG		
Liu, Yan-Ci et al., 2017 * [10]	9; 9	10	51.0 ± 7.1; 48.8 ± 11.7	50.8 ± 13.5	8/1; 8/1	8/2	36.4; 36.2	49.8	NR	Community ambulator
Plummer, P. et al., 2022 [18]	17	19	54.4 ± 16.4	59.6 ± 14.5	10/7	9/10	8.8	6.8	High fall risk	Community ambulator
Iqbal, M. et al., 2020 [13]	32	32	58.28 ± 7.131	58.87 ± 6.131	17/15	17/15	NR		High fall risk	Community ambulator
Yang, Yea Ru et al., 2007 [9]	13	12	59.46 ± 11.83	59.17 ± 11.98	7/6	7/5	48.96	56.16	NR	Community ambulator
Park, Myoung et al., 2019 [36]	15	15	56.30 ± 7.14	59.75 ± 7.75	11/9	13/7	21.67	21.45	Low fall risk	NR
Song, G.B. et al., 2015 [38]	20	20	55.37 ± 20.6	57.10 ± 7.83	9/11	12/8	14.75	14.30	Moderate fall risk	NR
Choi, J.H. et al., 2015 [3]	10	10	64.8 ± 10.5	54.6 ± 11.8	6/4	6/4	0.76	0.77	NR	NR
Hong, Su-Yeon et al., 2020 [31]	8	9	56.63 ± 8.78	66.22 ± 11.55	6/2	4/5	19	15.33	High fall risk	Home ambulator
Aydogdu, Y.T. et al., 2018 [39]	25	28	69.28 ± 5.03	71.21 ± 4.92	19/9	20/5	Chronic stroke		Low fall risk	NR
Shim, S. et al., 2012 [37]	17	16	65.59 ± 5.81	61.56 ± 6.17	12/5	8/8	16.29	17.44	NR	Community ambulator
Kim, Keun Jo et al., 2018 [35]	13	13	52.62 ± 9.84	56.15 ± 10.82	8/5	7/6	12.62	11.46	NR	Home ambulator
Kim, Hyeon Ae et al., 2013 [34]	14	15	57.1 ± 10.5	55.7 ± 14.1	9/5	10/5	7.2	6.8	NR	Home ambulator
Kannan, L. et al., 2019 [32]	10	10	57.5 ± 8.04	61 ± 4.6	7/6	6/5	106.8	109.08	Moderate fall risk	NR
Kim H et al., 2015 [33]	20	20	51.0 ± 13.5	52.1 ± 7.5	12/8	14/6	>6 month		NR	Community ambulator
Xing Wang et al., 2023 [29]	36	36	70.26 ± 3.54	71.13 ± 3.61	25/11	23/13	0.24	0.261	High fall risk	NR
CAI Qing et al., 2018 [30]	15	15	56.47 ± 3.48	58.07 ± 4.74	13/2	10/5	2.34	2.27	Moderate fall risk	NR
Zhang Qingmei et al., 2019 [28]	40	40	65.53 ± 3.69	66.73 ± 4.36	24/16	26/14	4.09	4.15	Low fall risk	NR
Baek, C.Y. et al., 2021 [19]	16	15	56.94 ± 8.79	56.13 ± 10.25	12/4	8/7	56.31	53.07	NR	Home ambulator
B. d. A. Antonio et al., 2023 [15]	13	13	54 ± 12.30	54 ± 12.30	7/6	6/7	70	67	Moderate fall risk	Community ambulator
L. L. Chuang et al., 2025 [16]	22	22	60.94 ± 10.06	59.21 ± 12.01	11/11	15/7	64.35	75.26	Low fall risk	Community ambulator
T. T. Yeh, K et al., 2023 [17]	22	20	63.55 ± 7.08	60.88 ± 12.28	19/3	16/4	63.68	52.10	High fall risk	Community ambulator

*: study with two experimental groups; EG: experimental group, CG: control group; NR: not reported.

**Table 2:** Characteristics of the intervention.

Study	Experimental Group Intervention	Types of Basic Motor Training	Control Group Intervention	Type of Control	Outcomes	Dual-Task Training Protocols
Liu, Yan-Ci et al., 2017 * [10]	CDT/MDT	Gait training	CT	Active	Gait speed, Stride length,	Participants in the CDT group performed cognitive tasks such as counting while walking; participants in the MDT group performed motor tasks such as holding balls while walking.
Plummer, P. et al., 2022 [18]	CDT	Gait training	STT	Passive	Gait speed, FMA_LE,	Participants performed two different cognitive tasks while walking.
Iqbal, M et al., 2020 [13]	MDT	Gait training	CT	Active	Gait speed, Stride length,	Dual task training included activities such as slowly walking backward, sideways, and forward on a smooth surface while holding a 100 g sandbag.
Yang, Yea-Ru et al., 2007 [9]	MDT	Gait training	No training	Passive	Stride length	Participants walked while manipulating either 1 or 2 balls.
Park, Myoung et al., 2019 [36]	CDT	Balance training	CT	Active	BBS	Various cognitive tasks relating to memory and attention were performed while maintaining balance.
Song, G.B. et al., 2015 [38]	MDT	Balance training	STT	Passive	BBS	Participants threw balls while standing on a balance pad.
Choi, J.H. et al., 2015 [3]	CDT + CT	Balance training	STT + CT	Active	FMA_LE	Participants used BioRescue to carry out simultaneous balance and cognitive training.
Hong, Su-Yeon et al., 2020 [31]	CDT	Balance training	STT	Passive	Stride length, BBS	A dual task of balance and cognition using traffic signals, a familiar form to the subjects, was applied as a program.
Baek, C.Y. et al., 2021 [19]	CDT + CT	Gait training	STT + CT	Passive	Gait speed, Stride length	Participants performed cognitive tasks comprising mental tracking, verbal fluency, and executive function while walking on the treadmill.
Aydogdu, Y.T. et al., 2018 [39]	CDT + CT	Gait training	STT + CT	Passive	BBS	Participants performed cognitive tasks such as counting and spelling while walking.
Shim, S. et al., 2012 [37]	MDT + CT	Gait training	CT	Passive	Gait speed, Stride length	Participants manipulated the ball while walking.
Kim, Keun-Jo et al., 2018 [35]	CDT	Gait training	STT	Passive	Gait speed, Stride length	Participants performed cognitive tasks such as memorizing and arithmetic subtraction while walking on a treadmill.
Kim, HyeonAe et al., 2013 [34]	MDT + CT	Gait training	STT + CT	Passive	Gait speed, Stride length	Participants were asked to hold sandbags while walking on a flat surface.
Kannan, L. et al., 2019 [32]	CDT	Mixed training	CT	Active	BBS	Participants performed Wii-fit games in conjunction with cognitive tasks.
Kim H et al., 2015 [33]	CDT + CT	Gait training	STT + CT	Passive	Gait speed, Stride length	Participants performed virtual cognitive dual-task treadmill training using a video recording.
Xing Wang et al., 2023 [29]	CDT + CT	Balance training	STT + CT	Passive	BBS, FMA_LE	Participants performed cognitive tasks such as counting while using the PK254 balance system for training.
CAI Qing et al., 2018 [30]	MDT + CT	Gait training	STT + CT	Passive	BBS, FMA_LE	Participants were asked to throw and move objects while walking.
Zhang Qingmei et al., 2019 [28]	MDT + CT	Gait training	STT + CT	Passive	FMA_LE	Participants held different items, such as basketballs or sandbags, while walking on the treadmill.
B. d. A. Antonio et al., 2023 [15]	CDT	Mixed training	STT	Passive	Gait speed	Participants performed cognitive tasks such as calculation and memorization while carrying out resistance and aerobic training.
L. L. Chuang et al., 2025 [16]	CDT	Mixed training	STT	Passive	Gait speed	Participants performed visual discrimination, verbal fluency, and calculation while carrying out multimodal training.
T. T. Yeh, K et al., 2023 [17]	CDT	Bike training	CT	Active	Gait speed	Participants engaged in simultaneous stationary bike cycling and computer-based cognitive training.

*: study with two experimental groups; CT: conventional training; CDT: cognitive dual-task training; MDT: motor dual-task training; STT: single-task training.

**Table 3:** PEDro scale.

Study	Eligibility Criteria	Random Allocation	Concealed Allocation	Baseline Comparability	Blind Subjects	Blind Therapists	Blind Assessors	Adequate Follow-Up	Intention-to-Treat	Between-Group Comparisons	Point Estimates and Variability	Total
Liu, Yan-Ci et al., 2017 [10]	1	1	1	1	0	0	0	1	1	1	1	7/10
Plummer, P. et al., 2022 [18]	1	1	1	1	0	0	1	1	1	1	1	8/10
Iqbal, M. et al., 2020 [13]	1	1	1	1	0	0	0	1	1	1	1	7/10
Yang, YeaRu et al., 2007 [9]	1	1	1	1	0	0	1	1	0	1	1	7/10
Park, Myoung et al., 2019 [36]	1	1	1	1	0	0	1	1	1	1	1	8/10
Song, G.B. et al., 2015 [38]	1	1	0	1	0	0	0	1	1	1	1	6/10
Choi, J.H. et al., 2015 [3]	1	1	0	1	0	0	0	1	1	1	1	6/10
Hong, Su-Yeon et al., 2020 [31]	1	1	0	1	0	0	0	1	1	1	1	6/10
Baek, C.Y. et al., 2021 [19]	1	1	1	1	0	0	1	1	1	1	1	8/10
Aydogdu, Y.T. et al., 2018 [39]	1	1	0	1	0	0	0	0	1	1	1	5/10
Shim, S. et al., 2012 [37]	1	1	0	1	0	0	0	1	1	1	1	6/10
Kim, K.J et al., 2018 [35]	1	1	1	1	0	0	0	1	1	1	1	7/10
Kim, HyeonAe et al., 2013 [34]	1	1	0	1	0	0	0	1	1	1	1	6/10
Kannan, L. et al., 2019 [32]	1	1	0	1	0	0	0	1	1	1	1	6/10
Kim H et al., 2015 [33]	1	1	0	1	0	0	0	1	1	1	1	6/10
Xing Wang et al., 2023 [29]	1	1	0	1	0	0	0	0	1	1	1	5/10
CAI Qing et al., 2018 [30]	1	1	0	1	0	0	1	0	1	1	1	6/10
Zhang Qingmei et al., 2019 [28]	1	1	0	1	0	0	1	0	1	1	1	6/10
B. d. A. Antonio et al., 2023 [15]	1	1	0	1	0	0	1	0	0	1	0	5/10
L. L. Chuang et al., 2025 [16]	1	1	0	0	0	0	1	1	0	1	0	5/10
T. T. Yeh, K et al., 2023 [17]	1	1	0	1	0	0	1	1	0	1	0	6/10

## Data Availability

The data presented in this study are available in the Supplementary Materials.

## Supplementary Materials

The following supporting information can be downloaded at: https://www.mdpi.com/article/10.3390/geriatrics11030065/s1, Supplementary Table S1: Search Strategy; Supplementary Table S2: GRADE Quality Assessment of Evidence; raw data.xlsx; PRISMA_2020_checklist.

## Abbreviations

The following abbreviations are used in this manuscript:

MDT	Motor Dual-Task Training
CDT	Cognitive Dual-Task Training
ADL	Activities of Daily Living
BBS	Berg Balance Scale
FMA_LE	Fugl–Meyer Lower Extremity Assessment Score

## Appendix A

### Appendix A.2. Publication Bias

**Table A1:** Publication bias results of Egger’s test. All *p*-values were above 0.05, indicating no significant publication bias.

Outcome	Coef	Std. Err	95% CI	t Value	*p* Value
Gait speed	−0.16	4.24	−9.49 to 9.18	−0.04	0.97
Stride length	−2.9	3.14	−10.33 to 4.53	−0.92	0.39
BBS	1.79	3.3	−6.68 to 10.26	0.54	0.61
FMA_LE	0.16	3.37	−10.57 to 10.89	0.05	0.97
