# Supplementary material for: Differential Effects of Cognitive vs. Motor Dual-Task Training in Stroke Rehabilitation: A Precision-Focused Meta-Analysis
Source: Geriatrics (Basel). 2026 May 31;11(3):65. doi: 10.3390/geriatrics11030065 (PMC13300573; doi:10.3390/geriatrics11030065)
Supplement: Supplementary file 1 [file geriatrics-11-00065-s001.zip › Table S1.pdf]

Database	Search Strategy String
Web of Science	TS=(<br>(stroke* OR "cerebrovascular accident*" OR CVA<br>OR "brain vascular accident*" OR "cerebrovascular apoplexy")<br>AND<br>("dual task" OR "dual-task" OR "dual task training" OR "dual-task training"<br>OR "dual task interference" OR "dual-task interference"<br>OR "dual task performance" OR "dual-task performance"<br>OR "divided attention" OR "task interference")<br>)
PubMed	("Stroke"[Mesh] OR "Cerebral Hemorrhage"[Mesh] OR "Brain Ischemia"[Mesh]<br>OR stroke*[tiab] OR "cerebrovascular accident*"[tiab] OR CVA[tiab]<br>OR "brain vascular accident*"[tiab] OR "cerebrovascular apoplexy"[tiab]) AND ("dual task"[tiab] OR "dual-task"[tiab] OR "dual task training"[tiab]<br>OR "dual-task training"[tiab] OR "dual task interference"[tiab]<br>OR "dual-task interference"[tiab] OR "dual task performance"[tiab]<br>OR "dual-task performance"[tiab] OR "divided attention"[tiab]<br>OR "task interference"[tiab])
Medline	((exp Stroke/ OR exp Cerebral Hemorrhage/ OR exp Brain Ischemia/<br>OR (stroke* OR "cerebrovascular accident*" OR CVA OR "brain vascular accident*" OR "cerebrovascular apoplexy").ti,ab.)<br>AND<br>((dual task OR dual-task OR dual task training OR dual-task training<br>OR dual task interference OR dual-task interference<br>OR dual task performance OR dual-task performance<br>OR divided attention OR task interference).ti,ab.)
Embase	((exp stroke/ OR exp cerebrovascular accident/ OR exp brain infarction/<br>OR exp intracerebral hemorrhage/<br>OR (stroke* OR "cerebrovascular accident*" OR CVA OR "brain vascular accident*" OR "cerebrovascular apoplexy").ti,ab.)<br>AND<br>((dual task OR dual-task OR dual task training OR dual-task training<br>OR dual task interference OR dual-task interference<br>OR dual task performance OR dual-task performance<br>OR divided attention OR task interference).ti,ab.)
Cochrane Library	((MeSH descriptor: [Stroke] explode all trees<br>OR MeSH descriptor: [Cerebral Hemorrhage] explode all trees<br>OR (stroke* OR "cerebrovascular accident*" OR CVA<br>OR "brain vascular accident*" OR "cerebrovascular apoplexy"):ti,ab,kw)<br>AND<br>("dual task" OR "dual-task" OR "dual task training" OR "dual-task training"<br>OR "dual task interference" OR "dual-task interference"<br>OR "dual task performance" OR "dual-task performance"<br>OR "divided attention" OR "task interference":ti,ab,kw)
CINAHL	((MH "Stroke+" OR MH "Cerebral Hemorrhage+"<br>OR stroke* OR "cerebrovascular accident*" OR CVA<br>OR "brain vascular accident*" OR "cerebrovascular apoplexy")<br>AND<br>("dual task" OR "dual-task" OR "dual task training"

	OR "dual-task training" OR "dual task interference"<br>OR "dual-task interference" OR "dual task performance"<br>OR "dual-task performance" OR "divided attention"<br>OR "task interference")
Chinese National Knowledge Infrastructure	(主题 = (脑卒中 OR 卒中 OR 脑血管意外 OR 中风))<br>AND<br>(主题 = (双任务 OR 双重任务 OR 双任务训练 OR 双重任务训练<br>OR 双任务干扰 OR 双任务表现 OR 注意分配))

- The literature search commenced in November 2023.
- No filters were applied in any database to maximize the inclusion of potentially relevant studies. Duplicate records and ineligible study types were subsequently removed using EndNote.
- The direct English translation of search strategy string for Chinese National Knowledge Infrastructure is:
  (Subject = (stroke OR cerebrovascular accident OR CVA))
  AND
  (Subject = (dual task OR dual-task OR dual-task training
  OR dual-task interference OR dual-task performance
  OR attention allocation))
