# Supplementary material for: Differential Effects of Cognitive vs. Motor Dual-Task Training in Stroke Rehabilitation: A Precision-Focused Meta-Analysis
Source: Geriatrics (Basel). 2026 May 31;11(3):65. doi: 10.3390/geriatrics11030065 (PMC13300573; doi:10.3390/geriatrics11030065)
Supplement: Supplementary file 1 [file geriatrics-11-00065-s001.zip › Table S2.pdf]

GRADE Quality Assessment of Evidence

Gait speed						
Study	Risk of Bias	Inconsistency	Indirectness	Imprecision	Publication	Certainty of Evidence
Liu, Yan-Ci 2017*	Some concerns	Low	Low	Serious (small sample)	Low	Moderate
Plummer, P. 2022	Low	Low	Low	Some concerns	Low	High
Iqbal, M.2020	Serious (no blinding)	Low	Low	Low	Low	Moderate
Baek, C. Y. 2021	Low	Low	Low	Low	Low	High
Shim, S.2012	Some concerns	Low	Low	Some concerns	Low	Moderate
Kim, Keun-Jo2018	Serious (no blinding)	Low	Low	Low	Low	Moderate
Kim, HyeonAe2013	Serious (no blinding)	Low	Low	Some concerns	Low	Low
Kim H2015	Serious (no blinding)	Low	Low	Low	Low	Moderate
B. d. A. Antonio 2023	Low	Low	Low	Serious (small sample)	Low	Moderate
L. L. Chuang 2025	Low	Low	Low	Low	Low	High
T. T. Yeh, K 2023	Low	Low	Low	Low	Low	High

Stride Length						
Study	Risk of Bias	Inconsistency	Indirectness	Imprecision	Publication	Certainty of Evidence
Liu, Yan-Ci 2017*	Some concerns	Low	Low	Serious (wide standard deviations)	Low	Moderate
Iqbal, M.2020	Serious (no blinding)	Low	Low	Low	Low	Moderate
Yang, Yea-Ru2007	Low	Low	Low	Some concerns	Low	High
Hong, Su-Yeon2020	Serious (no blinding)	Low	Low	Some concerns	Low	Moderate
Baek, C. Y. 2021	Low	Low	Low	Low	Low	High
Shim, S.2012	Low	Low	Low	Some concerns	Low	High
Kim, Keun-Jo2018	Serious (no blinding)	Low	Low	Low	Low	Moderate
Kim, HyeonAe2013	Serious (no blinding)	Low	Low	Some concerns	Low	Low
Kim H2015	Serious (no blinding)	Low	Low	Serious (huge baseline difference)	Low	Low

FMA						
Study	Risk of Bias	Inconsistency	Indirectness	Imprecision	Publication	Certainty of Evidence
Plummer, P. 2022	Some concerns	Low	Low	Some concerns	Low	Moderate
Choi, J. H.2015	Low	Low	Low	Low	Low	High
Xing Wang 2023	Serious (no blinding)	Low	Low	Low	Low	Moderate
CAI Qing2018	Some concerns	Low	Low	Some concerns	Low	Moderate
Zhang Qingmei 2019	Some concerns	Low	Low	Low	Low	High

BBS						
Study	Risk of Bias	Inconsistency	Indirectness	Imprecision	Publication	Certainty of Evidence
Park, Myoung-Ok2019	Some concerns	Low	Low	Some concerns	Low	Moderate
Song, G. B.2015	Serious (no blinding)	Low	Low	Serious (wide standard deviations)	Low	Low
Hong, Su-Yeon2020	Serious (no blinding)	Low	Low	Low	Low	Moderate
Aydogdu, Y. T.2018	Serious (self-selection bias)	Low	Low	Some concerns	Low	Moderate
Kannan, L.2019	Low	Low	Low	Low	Low	High
Xing Wang 2023	Serious (no blinding)	Low	Low	Low	Low	Moderate
CAI Qing2018	Some concerns	Low	Low	Some concerns	Low	Moderate
